# Psychosocial interventions for suicidal and self-injurious-related behaviors among adolescents: a systematic review and meta-analysis of Chinese practices

**DOI:** 10.3389/fpubh.2023.1281696

**Published:** 2023-12-18

**Authors:** Junjie Lu, Wanting Gao, Zexin Wang, Nan Yang, Weng Ian Phoenix Pang, Grace Ka In Lok, Wenwang Rao

**Affiliations:** ^1^Department of Preventive Medicine, Shantou University Medical College, Shantou, Guangdong, China; ^2^Faculty of Health Sciences and Sports, Macao Polytechnic University, Macao, Macao SAR, China; ^3^Macao Polytechnic University, Peking University Health Science Center-Macao Polytechnic University Nursing Academy, Macao, Macao SAR, China

**Keywords:** psychosocial intervention, suicide, self-injurious behavior, adolescent, China

## Abstract

**Background:**

Suicidal and self-injurious-related behaviors (SSIRBs) are a serious public health challenge in China. However, a comprehensive systematic review of psychosocial interventions for SSIRBs among Chinese adolescents has not been performed. To fill this gap, this systematic review and meta-analysis aimed to examine psychosocial interventions for SSIRBs among Chinese adolescents.

**Methods:**

Eight international (PubMed, EMBASE, Cochrane Library, ScienceDirect, Clinical Trial, CINAHL, PsycINFO, and Web of Science) and four Chinese (Wanfang, SinoMed, CEPS, and CNKI) databases were searched from inception to 31 January 2023. Data extraction and quality assessment were independently conducted by two groups of researchers. Qualitative synthesis and meta-analysis were both used.

**Results:**

The initial search yielded 16,872 titles. Of the 649 full texts reviewed, 19 intervention articles focusing on SSIRBs met the inclusion criteria. Thirteen out of the 19 included studies involved cognitive–behavioral therapy (CBT). Seven non-suicidal self-injury (NSSI) studies assessing self-injurious behaviors were included (six short-term studies and three long-term studies). Compared with long-term interventions [−1.30 (95% CI: –1.84, −0.76)], short-term psychosocial interventions had a higher standardized mean difference (SMD) value [1.86 (95% CI: –2.72, −0.99)]. Meta-regression showed an inverse relationship between the treatment response and sample size (slope = 0.068, *Z* = 2.914, *p* = 0.004) and proportion of females (slope = 1.096, *Z* = 5.848, *p* < 0.001). Subgroup analyses showed that compared with the “less than 1 month” group [−0.494 (−0.783, −0.205)], in the “immediate postintervention” group, the pooled estimate was significantly lower [−2.800 (−4.050, −1.550), *p* < 0.001].

**Conclusion:**

Our review systematically summarized the key characteristics and effectiveness of existing psychosocial interventions for SSIRBs among Chinese adolescents. Short-term psychosocial interventions for NSSI were significantly effective in reducing self-injurious behavior scores, especially in the immediate postintervention period. More favorable treatment responses could be observed in both male and small samples.

## 1 Introduction

Approximately 800,000 persons die globally each year due to suicide, among whom approximately 60,000 are young people (1, 2). Suicide is the main cause of adolescent death (1), and approximately 1% to 18% of adolescents each year are diagnosed with suicidal and self-injurious-related behaviors (SSIRBs) (3, 4), including non-suicidal self-injury (NSSI), self-injurious behavior (SIB), suicide ideation (SI), and suicide attempts (SAs) (5–11), some of which are recognized as stages of the suicide continuum (12). Suicidal behaviors range from SI, suicide plans (SPs), and SAs to completed suicide (13). SI is widely accepted as a reflection of engagement in suicide-related behaviors (11). SAs are defined as potential self-injurious behaviors associated with at least some intent to die (14). Manifested as the deliberate, self-induced destruction of body tissues, the definition of NSSI as an essential component of SIB is based on the absence of suicidal intent (15). As the main component of SSIRBs, which are a severe public health problem (1, 16), suicide-related behaviors often lead to serious adverse consequences (17). These results are mainly reflected in personal psychological and physical pain as well as negative impacts on families and even communities (18).

In light of the above, many psychosocial intervention studies have been conducted in Western countries (19–21). For example, two previous studies demonstrated that internet-based cognitive–behavioral therapy (e-CBT) could reduce SI and alleviate symptoms of depression and despair (20, 21). A program from the Youth Aware of Mental Health (YAMH) in Europe found that the use of CBT significantly reduced SI and SAs among high school students (19). However, high-quality psychosocial intervention studies are rarely conducted among Chinese adolescents with SSIRBs. Moreover, early reviews also showed that both CBT and dialectical behavior therapy (DBT) were effective in treating individuals with SSIRBs (22, 23). However, due to language restrictions, Chinese intervention studies were rarely included in early systematic reviews and meta-analyses.

The rapid process of socialization, as well as the unique Chinese traditional cultural background and policies (24, 25), have had some impact on changes in suicide rates among adolescents (26, 27). There is accumulated evidence that the prevalence rates and mortality of SSIRBs are on the rise (28–30). Effective interventions and strategies that are suitable for China’s national conditions urgently need to be developed and implemented. Previous studies have shown that evidence-based interventions in clinical settings are effective (31, 32), but there is still a lack of high-quality systematic reviews and meta-analyses to guide interventions for Chinese adolescents with SSIRBs. At present, only a systematic scoping review in the Chinese population summarized the prevalence of and risk factors and interventions for NSSI (16). Nevertheless, several disadvantages need to be noted: first, only six databases were searched; second, a meta-analysis of interventions for NSSI was not performed; and third, only studies relating to NSSI were included.

From the above, no study has conducted a comprehensive and systematic review of psychosocial interventions for SSIRBs in China. To the best of our knowledge, this is the first study to systematically summarize psychosocial interventions for SSIRBs among Chinese adolescents. It might help us develop more authoritative intervention methods in the selection of treatment for SSIRBs, especially in intervening in NSSI among adolescents in China.

## 2 Materials and methods

This meta-analysis was prospectively registered in the International Platform of Registered Systematic Review and Meta-analysis Protocols (INPLASY; registration number: 202350069) and was performed in accordance with the Preferred Reporting Items for Systematic Reviews and Meta-Analyses (PRISMA) statement.

### 2.1 Eligibility criteria and outcome measures

According to the PICOS tool, the inclusion criteria were as follows: Participants (P): Chinese adolescents (up to and 18 years old) with SSIRBs (e.g., NSSI, SI, and SAs); intervention (I): psychosocial treatment (e.g., CBT, counseling, and systemic interventions); comparison (C): non-intervention or non-experimental group intervention (e.g., routine treatment and drug therapy); outcomes (O): effectiveness; and study design (S): randomized-controlled trials (RCTs), clinical-controlled trials (CCTs), and prepost studies. The exclusion criteria included (a) studies of Chinese adolescents with mental diseases, (b) studies using qualitative methods, and (c) non-Chinese or non-English studies. The main outcomes were the mean and standard deviation (SD) of scores on the SSIRB scale, such as the Questionnaire for Middle School Students’ Behavior (QMSSB) and the Adolescent Non-suicidal Self-injury Assessment Questionnaire (ANSAQ). Secondary outcomes were the mean and SD of scores on other symptom scales relating to hopelessness, depression, anxiety, and family function, such as the Chinese Family Function Scale (CFFS) and Middle School Students Depression Scale (MSSDS).

### 2.2 Search strategy and study selection

A literature search in both international (PubMed, EMBASE, Cochrane Library, ScienceDirect, Clinical Trial, CINAHL, PsycINFO, and Web of Science) and Chinese (Wanfang, SinoMed, CEPS, and CNKI) databases from inception to 31 January 2023 was independently conducted by two groups of researchers (JJL, WTG, WWR and NY, ZXW, and KIG L). To identify studies for review (33), the following subject and free terms were used: (“auto mutilat*“OR “cutt*” OR “headbang*” OR “overdos*” OR “selfdestruct*” OR “selfharm*” OR “selfimmolat*” OR “selfinflict*” OR “selfinjur*” OR “selfpoison*” OR “suicid*” OR “suicide, attempted” OR “suicidal ideation”) AND (“adolescent” OR “teen” OR “youth” OR “teenager”) AND (“China” OR “Chinese”). More detailed information is provided in Supplementary Figure S1.

The same two groups of researchers independently screened the titles and abstracts and then read the full texts of relevant publications for eligibility. Any discrepancy was discussed with another researcher (WIP P). The reference lists of the included articles, relevant systematic reviews, and meta-analyses were searched manually for additional studies (1, 33–50).

### 2.3 Data extraction

A predesigned Excel data collection sheet was used by the two groups of researchers to independently extract relevant data, including the following characteristics of studies and participants: first author, year of survey and publication, survey province, study type, sampling method, sample size, types of interventions in the control and experimental groups, setting, inclusion criteria, intervention duration, type of SSIRB, age range, mean and SD of participant age, number and proportion of males, definitions of various types of SSIRBs, and measurements.

According to a categorical criterion of psychosocial intervention (51), types of intervention were clustered into a new parent set and a subset. Data were extracted and double-checked independently by two researchers (KIG L and WWR). Disagreements were settled through discussion with another senior researcher (WIP P). To address the missing SD values, we substituted them with the average SD values reported from other RCTs that assessed the same treatment or outcome measures (52). GetData Graph Digitizer version 2.25.0.32 was used to extract related information presented in images.

### 2.4 Quality assessment and evidence level

The quality of the included studies was evaluated by quality assessment checklists based on study designs. RCT studies were evaluated by the Jadad scale (0–5 points) (53). CCT studies were assessed using the National Heart, Lung, and Blood Institute (NHLBI) tailored tool for controlled intervention studies (0–16 points) (54). The NHLBI checklist developed for before-after studies with no control group was used for pre–post studies (0–12 points) (54). The quality assessment was conducted under the assumption that each criterion contributed equally to study quality. Study quality was independently assessed by two reviewers (WWR and JJL). Disagreements were resolved through discussions with another researcher (KIG L).

### 2.5 Statistical analysis

#### 2.5.1 Qualitative synthesis

Based on a qualitative analysis method, we synthesized the study traits, intervention attributes, and outcomes concerning the efficacy of the interventions.

#### 2.5.2 Meta-analysis

According to the duration of follow-up (55), studies were categorized into two periods in the meta-analysis: (1) short-term studies, which referred to studies in which the outcomes were assessed within 1 month after the intervention and (2) long-term studies, which encompassed studies in which the outcomes were evaluated at least 1 month after the intervention. Owing to the limitation of the number of included studies, more than three articles with the same SSIRBs and relevant self-injurious assessments were considered for meta-analysis. Given the different sampling methods, demographic characteristics, and instruments between studies, the estimates of self-injurious-related behavior scores were calculated as standardized mean differences (SMDs) using the Der Simonian and Laird random-effects model (56). Heterogeneity across studies was estimated with Cochran’s *Q* test and *I*^2^ statistics, with *I*^2^ ≥ 50% or a Cochran’s *Q* test value of *p* of <0.05 indicating significant heterogeneity (57). Subgroup analyses were conducted according to the following categorical variables: assessment period (“immediate postintervention” group vs. “less than 1 month” group), measurements [ANSAQ vs. QMSSB/Adolescent Self Harm Scale (ASHS) vs. others], definition of NSSI (DSM-V criteria vs. hospitalization vs. others), and geographic region (Eastern China vs. Western China, according to the National Bureau of Statistics of China). Meta-regression analyses were used to examine the associations between self-injurious-related behavior scores and the sample size, mean age, and sex ratio (female/male). Sensitivity analyses were conducted to explore the outlier studies. Funnel plots and Begg’s test were used to assess publication bias. The significance level was set at 0.05 (two-tailed). The meta-analyses were conducted using Comprehensive Meta-Analysis software, Version 2 (CMA, Biostat Inc., Englewood, New Jersey, USA) and RevMan software, Version 5.3.

## 3 Results

### 3.1 The overall characteristics of the included literature

**Figure 1:**
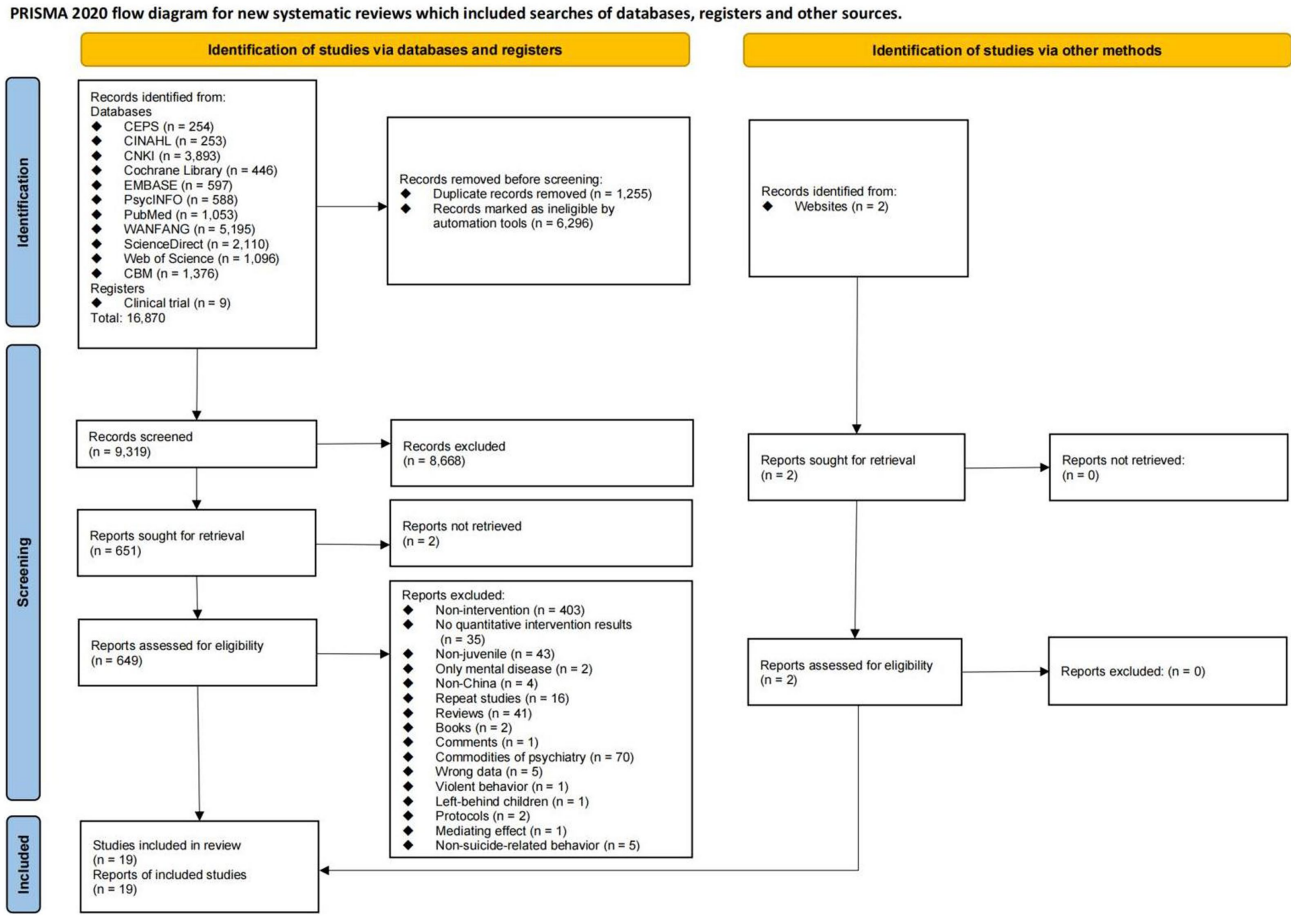
PRISMA flow diagram. From Page et al. (58).

Altogether, 16,872 articles were screened. Of these articles, only 9,319 were deemed eligible after filtering based on titles and abstracts. After full-text screening, 19 studies with 1,683 participants (1,060 subjects in the intervention group and 623 subjects in the control group) met the selection criteria (Figure 1). The publication time ranged from 2012 to 2022. No relevant literature from Hong Kong, Macao, or Taiwan was eligible, but the included studies were distributed in different provinces of mainland China (Table 1).

**Table 1:** Characteristics of included studies.

									**Control group**		**Experimental group**							
**No**	**First author**	**Study year**	**Publication year**	**Province**	**Study type**	**Grouping**	**Sample size A**	**Sample size B**	**Sample size B**	**Intervention type**	**Sample** **size B**	**Intervention** **type**	**Study site**	**Type of SSIRB**	**Definition** **Of SSIRB**	**Age (Mean ± SD)**	**Male N (%)**	**References**
1	Yang RL	2017–2019	2021	Guangdong	RCT	RNT	80	80	40	CHE	40	CHE + GD + MT	S	NSSI	NSSI episode ≥ 1.	13.40 ± 0.86	46 (57.50%)	(59)
2	Xie HX	2019–2020	2022	Zhejiang	RCT	RA	102	102	50	CT^%^	52	GST	H	NSSI	NSSI admitted to TSPH.	11.40 ± 1.29	49 (48.04%)	(60)
3	Wang YP	2021–2022	2022	Guangdong	RCT	RNT	96	86	42	CN	44	CBT-A	H	NSSI	NSSI: DSM-V.	15.59 ± 2.99	12 (13.95%)	(61)
4	Su XY	2019–2021	2022	Shanxi	RCT	RNT	90	90	45	CT	45	CT + DBT	H	NSSI	NSSI admitted to SFH.	16.25 ± 1.40	52 (57.78%)	(31)
5	Li L	NR	2012	Shanghai	RCT	Random^&^	210	210	109	CPC	101	CPC in School + CBT (Group)	S	SI + SA	(1) ST ≥ 5 days and/or having AS in the past year. (2) BDI ≥ 18.	15.60 ± 1.68	103 (48.05%)	(62, 63)
6	Huang J	2019–2020	2022	Jiangsu	RCT	RA	126	126	63	CBT	63	CA + CBT	H	NSSI	(1) NSSI: criterion A. (2) No SI.	16.65 ± 2.50	57 (45.24%)	(32)
**7**	Du WL	2019–2022	2022	Yunnan	RCT	RA	40	40	20	CN	20	SPN	H	NSSI	NSSI: DSM-V.	14.90 ± 3.95	0	(64)
8	Ding D	2019–2020	2021	Shandong	RCT	RNT	100	100	50	Fluoxetine +PSE	50	Fluoxetine +ORFT	H	NSSI	(1) First NSSI: DSM-V. (2) MHT ≥ 56.	14.96 ± 1.46	33 (33.00%)	(65)
9	Chen G	2021	2022	Guangdong	RCT	CP	92	92	46	CPI	46	TFP + ER	H	NSSI	NSSI admitted to ABHGMU.	15.19 ± 2.00	33 (35.87%)	(66)
10	Xia S	2021–2022	2022	Jiangxi	RCT	RNT	80	80	40	CT	40	CT + FPACT	H	NSSI	NSSI: criterion B.	15.58 ± 2.41	42 (52.50%)	(67)
11	Li JC	NR	2016	Hunan	Pre-post	NR	/	6	/	/	6	EMGC	S	SIB	ASIBS>7 and EES<30.	12.33 ± 0.52	6 (100.00%)	(68)
12	Liu JT	NR	2013	Shandong	Pre-post	NR	/	330	/	/	330	GPI	S	SI	SI: BSI-CV.	/	166 (50.30%)	(69)
13	Lin YT	NR	2019	Jiangsu	Pre-post	NR	/	1	/	/	1	SPT	S	SIB	SIB: ASIBS.	17	0	(70)
14	Chang XD	NR	2015	Shanghai	Pre-post	NR	/	48	/	/	48	SPI	S	SI	SIOSS ≥ 12 or DSMSS ≥ 3.	/	/	(71)
15	Xie HT	NR	2014	Shanghai	Pre-post	NR	/	56	/	/	56	CPC + CBPC (group) +SPE (parents)	S	SB + SI	(1) SI of BDI ≥ 1 and BDI ≥ 14. (2) Having ST or SA in the last 1 year.	15.11 ± 1.50	27 (48.21%)	(72)
16	Xue YW	NR	2022	Anhui	CCT	NR	32	32	16	NR	16	GC	S	SIB	SIB experiences.	NR	16 (50.00%)	(73)
17	Xue Y	2020	2022	Chongqing	CCT	NR	120	120	60	CT (DT/CBT)	60	CT + FPACT	H	NSSI	NSSI admitted to CMHC.	14.45 ± 1.62	69 (57.50%)	(74)
18	Rong J	2019	2020	Sichuan	CCT	AN	64	64	32	CN + DT	32	PN + CN + DT	H	NSSI	NSSI admitted to FPHC.	14.07 ± 2.05	36 (56.25%)	(75)
19	Li BC	NR	2016	Jiangxi	RCT	RA	20	20	10	SGC	10	ER (group)	S	SIB	SIB (middle degree + in the past month)	15.75 ± NR	8 (40.00%)	(76)

Sample size A: refer to the total number of people after recruitment and before randomization. Sample size B: refer to the number after intervention. Criterion A – quoted from (77); criterion B – quoted from (78); ^&^, Based on class; ^%^, Sertraline combined with low-dose quetiapine or aripiprazole; ABHGMU, The affiliated brain hospital of Guangzhou medical university; AN, Arbitrary number; ASIBS, Adolescent self-injury behavior scale; BSI-CV, Beck scale for suicide ideation-Chinese version; CA, Colaizzi analysis; CBT, Cognitive behavior therapy; CBT-A, Cognitive behavior therapy for adolescent; CBPC, Cognitive behavior practice counseling; CCT, Clinical controlled trial; CMHC, The Chongqing mental health center; CN, Conventional nursing; CPI, Conventional psychological intervention; CP, Coin flipping; CHE, Conventional health education; CPC, Conventional psychological course; CT, Conventional therapy; DT, Drug therapy; DBT, Dialectical behavior therapy; DSMSS, Depression scale for middle school students; DSM-V, Diagnostic and statistical manual of mental disorders (Fifth edition); ER, Emotional regulation; EMGC, Emotional management group counseling; FPACT, Family participatory acceptance and commitment therapy; FPHC, The fourth people’s hospital of Chengdu; FPHN, The first people’s hospital of Nantong; FAHNU, The first affiliated hospital of Nanchang university; GC, Group counseling; GD, Gratitude diary; GPI, Group psychological intervention; GST, Group skill training; H, Hospital; MT, Mindfulness therapies; NSSI, Non-suicidal self-injury; MHT, Mental Health Test; ORFT, Object relations family therapy; PSE, Psychological education; PN, Psychological Nursing; RA, Random allocation; RCT, Randomized controlled trial; RNT, Random number table; S, School; SA, Suicide attempt; SB, Suicidal behavior; SIB, Self-injurious behavior; STFA, The Scale of Tendency to Forgive for Adolescents; SI, Suicidal ideation; ST, Suicidal thoughts; SPE, Suicide prevention education; SPT, Sand play therapy; SPI, Systemic psychological intervention; SPN, Strengthening psychological nursing; SFH, The Shanxi Fenyang hospital; SGC, Self-confidence group coaching; TFP, Transference-focused psychotherapy; TSPH, The Taizhou second people’s hospital; TPHF, The third people’s hospital of Foshan.

### 3.2 Suicidal-related behaviors

#### 3.2.1 Intervention targets

**Figure 2:**
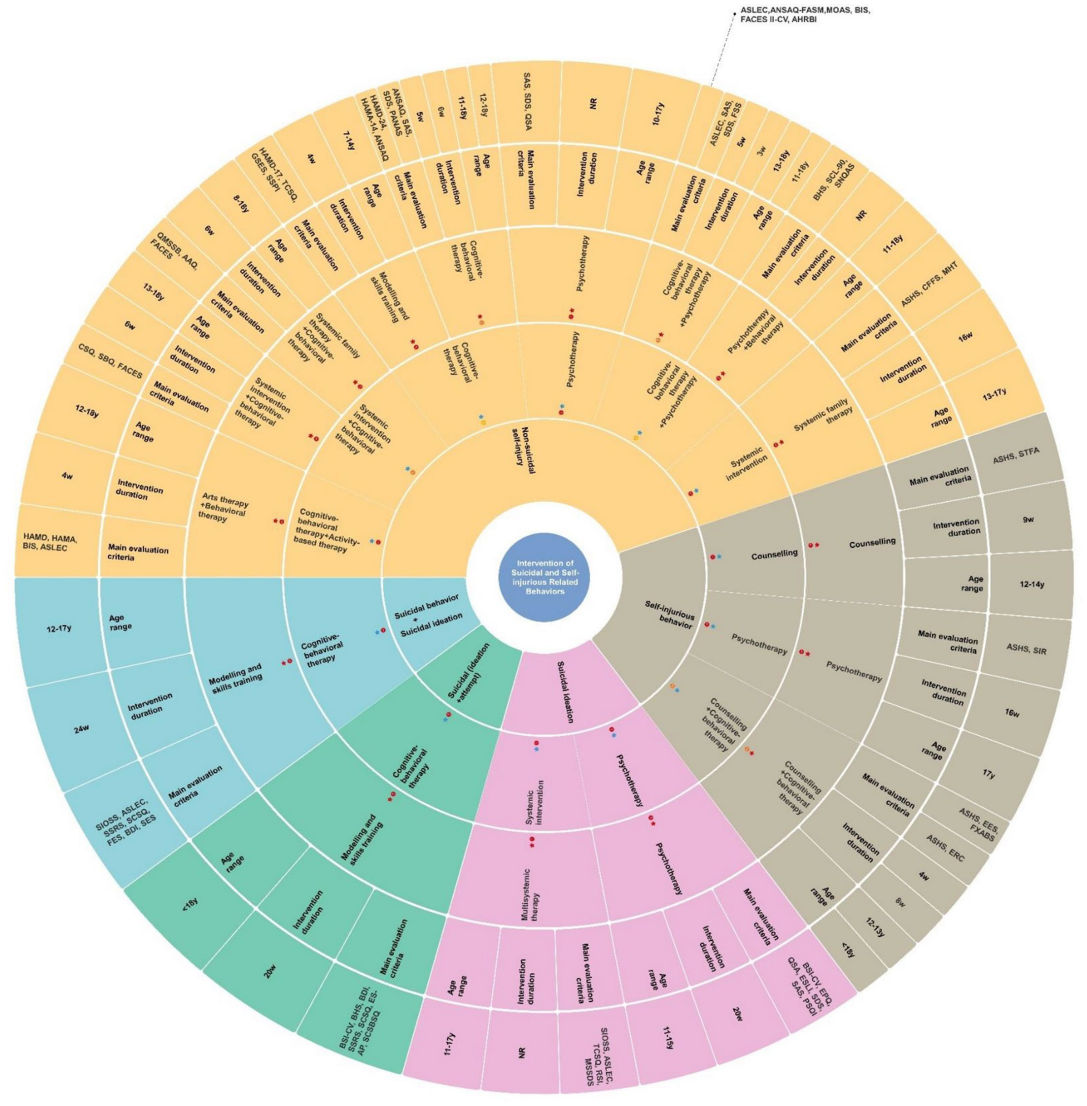
Intervention of suicidal and self-injurious related behaviors. 1 months = 4 weeks. Borders marked with “
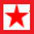
” belong to the large category of therapeutic approaches, while sub-category are tagged as “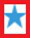.” 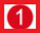: means that there is one study. 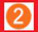: means that there are two studies. 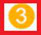: means that there are three studies. ASLEC, Adolescent Self-rating Life Events Check List; AHRBI, Adolescent Health Related Risk Behavior Inventory; ASHS, Adolescent Self Harm Scale; AAQ, Acceptance and Action Questionnaire; ANSAQ, Adolescent Non-suicidal Self-injury Assessment Questionnaire; BHS, Beck Hopelessness Scale; BDI, Beck Depression Inventory; BSI-CV, Beck Scale for Suicide Ideation-Chinese Version; BIS, Barratt Impulsiveness Scale; CSQ, Coping Style Questionnaire; CFFS, Chinese Family Function Scale; MSSDS, Middle School Students Depression Scale; RSI, Rate of Suicide Ideation; ES-AP, Emotional Skills Assessment Process; EPQ, Eysenck’s Personality Questionnaire; ESLI, Emotional–Social Loneliness Inventory; EES, Emotional Expression Scale; ERC, Emotional regulation scale; FES, Family Environment Scale; FASM, Functional Assessment of Self ⁃ mutilation; GSES, General Self-Efficacy Scale; FXABS, Fan Xiaodong Affect Balance Scales; FSS, Frequency and Severity of Self-injury; FACES, Family Adaptability and Cohesion Evaluation Scale; HAMD-24, Hamilton Depression Scale 24-item; HAMA-14, Hamilton Anxiety Scale 14-item; MOAS, Modified Overt Aggression Scale; PANAS, Positive and Negative Affect Scale; PSQI, Pittsburgh Sleep Quality Index; QSA, Suicide Attitude Questionnaire; QMSSB, Questionnaire for Middle School Students’ Behavior; SSRS, Social Support Rating Scale; SCSQ, Simplified Coping Style Questionnaire; SSPI, Scale of Social function in Psychosis Inpatients; SIOSS, Self-rating Idea of Suicide Scale; SES, Self-Esteem Scale; SAS, Self-rating Anxiety Scale; SDS, Self-rating Depression Scale; SCL-90, Symptom Check List-90; SNQAS, Self-developed Nursing Quality Assessment Scale; SCSBSQ, Self-compiled Suicide Behavior Survey Questionnaire; SBQ, Self-injury Behavior Questionnaire; SS, Self-esteem Scale; SIR, Self-Injury Rate; STFA, Scale of Tendency to Forgive for Adolescents; TCSQ, Trait Coping Style Questionnaire.

As shown in Figure 2, two studies focused on SI. Moreover, one study explored SI in conjunction with SAs. Another study examined SI combined with suicidality.

#### 3.2.2 Intervention area, year, and site

All four studies were geographically clustered in the eastern region of China. One study of an intervention for suicide-related behaviors was published every year. From 2012 to 2015, one article was retrieved for each year. All studies were performed at schools (Table 1).

#### 3.2.3 Intervention approaches

All empirical studies used group therapy. In addition, as shown in Figure 2, three strategies (i.e., systemic interventions, psychotherapy, and CBT) were mentioned in the included studies, and CBT appeared in two studies.

#### 3.2.4 Study quality

The majority of the three pre–post studies scored approximately 8 points out of a maximum score of 12 points. One article used a CCT design, which obtained 7 of 16 points (Supplementary Table S1).

### 3.3 Self-injurious behaviors

#### 3.3.1 General self-injury

Between 2016 and 2022, four studies explored intervention strategies for self-injurious behavior (Figure 2). Distributed in the eastern and central parts of China, these studies were conducted in Hunan Province, Jiangxi Province, Anhui Province, and Jiangsu Province. All studies were also carried out at schools. Apart from one study that used individual therapy alone, the other three studies applied group therapy. Half of the studies used combination therapy (i.e., counseling and CBT). The two other studies used counseling and psychotherapy. One study adopted a CCT design (7 points). The score of another RCT study, which did not use the appropriate randomization sequence method, was 1 point. Additionally, the average score of study quality in two pre- and postintervention studies was 7 points.

#### 3.3.2 Non-suicidal self-injury

##### 3.3.2.1 Intervention targets

Eleven studies focusing on NSSI were included in our review. Since four studies did not perform self-injury behavior assessments, seven studies were included in the final meta-analysis. Ultimately, six short-term studies and three long-term studies were pooled and analyzed according to predefined criteria. Two of the included studies used the ANSAQ-behavioral questionnaire, followed by the ASHS in one study, the QMSSB in one study, the FASM-behavioral questionnaire in one study, the SBQ in one study, and the Self-developed Nursing Quality Assessment Scale (SNQAS)-Self-injury risk assessment in one study. The detailed self-injury-related measurements and psychosocial intervention strategies are summarized in Figure 2.

##### 3.3.2.2 Intervention area, year, and site

Six studies were geographically concentrated in coastal cities, namely, Guangdong Province, Zhejiang Province, Jiangsu Province, and Shandong Province. Two studies were conducted in Central China, while another three studies were conducted in Western China. Only one study was published in 2020, and two studies were published in 2021. Then, most notably, the number of studies increased to eight in 2022, indicating an enormous growth trend of research interest. Furthermore, 10 studies were conducted in hospitals, with one study including a school sample.

##### 3.3.2.3 Intervention approaches

Three studies applied family therapy to treat adolescents with NSSI. The most commonly used psychological intervention was CBT, which was used in six studies.

##### 3.3.2.4 Study quality

Nine studies were classified as RCTs. The scores of most studies were greater than or equal to 2, but most studies did not use a double-blind method. Two studies utilized a CCT design, with an average score of 7. Overall, the study quality was acceptable (Supplementary Table S1).

##### 3.3.2.5 Rating of outcomes in the included studies (short-term)

The risk of self-injury and functional scores of the adolescents in the experimental group significantly decreased and were lower than those of the adolescents in the control group (31, 32, 64, 65, 67, 74). Only one study used the ANSAQ (experimental group: 58.68 ± 8.67 vs. control group: 68.61 ± 10.57, *p* < 0.001) (31). Using systematic interventions, CBT, and psychotherapy alone or in combination, four studies reported not only a significantly reduced risk of self-injury among adolescents but also significantly increased scores for family intimacy and adaptability (32, 65, 67, 74). Su et al. showed that CBT could significantly improve the scores of the Self-rating Anxiety Scale (SAS) (*Z* = 2.171, *p* = 0.030), Self-rating Depression Scale (SDS) (*Z* = 2.285, *p* = 0.022), and Positive and Negative Affect Scale (PANAS) [positive emotions: (experimental group: 26.39 ± 2.86 vs. control group: 22.23 ± 2.63, *p* < 0.001); negative emotions: (experimental group: 29.91 ± 2.73 vs. control group: 33.01 ± 3.19, *p* < 0.001)] (31).

##### 3.3.2.6 Rating of outcomes in the included studies (long-term)

There was a significant decrease in the risk of self-injury and impairment in functioning among adolescents who received psychological interventions compared to adolescents in the control group (31, 61, 65). Two studies reported significant improvements in depressive symptoms and anxiety symptoms after the intervention (31, 61). One study conducted by Ding’s team evaluated the efficacy at multiple time points after the intervention (65). The findings indicated that the self-injury scores in the experimental group significantly decreased (8 weeks: 14.5 ± 3.92 vs. 12 weeks: 13.32 ± 4.04 vs. 16 weeks: 8.62 ± 4.05).

##### 3.3.2.7 Meta-analysis

###### 3.3.2.7.1 Effectiveness in the short term

**Figure 3:**
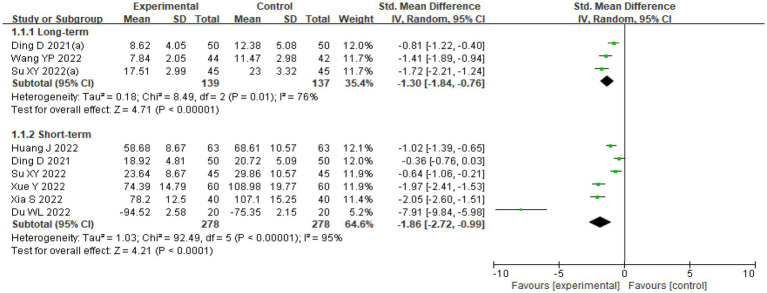
Effectiveness of interventions targeting NSSI. Ding D 2021(a) means the evaluation period: 16 weeks after intervention. Su XY 2022(a) means the evaluation period: 6 weeks after intervention.

**Figure 4:**
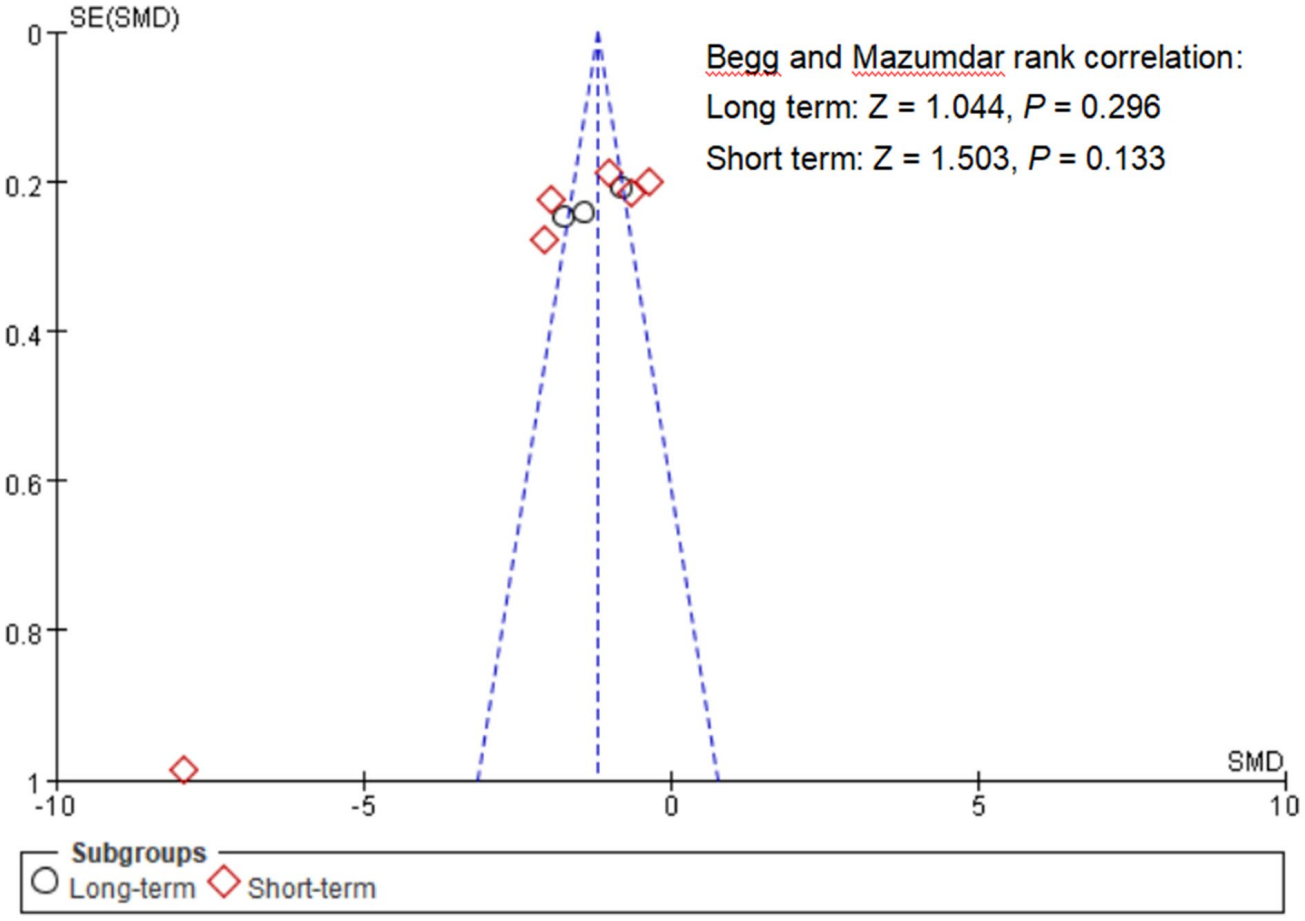
Funnel plot of publication bias. The *x*-axis represents the SMD for each study, while the *y*-axis is the standard error of the SMD. The dashed line represents the 95% confidence interval (CI).

The combined SMD value of self-injurious behavior scores was −1.86 (95% CI: –2.72, − 0.99). Considerable heterogeneity was found (*I^2^* = 95%, *p* < 0.001, Figure 3). However, no publication bias was found (Begg’s test = 1.503, *p* = 0.133, Figure 4). Based on sensitivity analysis, it was determined that the pooled SMD value of psychological interventions remained stable regardless of the exclusion of any single study (Supplementary Figure S2). The results of subgroup analyses using assessment periods showed that compared with the “less than 1 month” group [−0.494 (−0.783, −0.205)], in the “immediate postintervention” group, the pooled estimate was lower [−2.800 (−4.050, −1.550)]. As the sample size (slope = 0.068, *Z* = 2.914, *p* = 0.004) and proportion of females (slope = 1.096, *Z* = 5.848, *p* < 0.001) decreased, a more favorable treatment response was indicated (Supplementary Table S2).

###### 3.3.2.7.2 Effectiveness in the long term

The pooled SMD value of self-injurious behavior scores was −1.30 (95% CI: –1.84, − 0.76), indicating relatively poor efficacy. The presence of pronounced heterogeneity was noted (*I^2^* = 76%, *p* = 0.01, Figure 3). Publication bias was not found (Begg’s test = 1.044, *p* = 0.296, Figure 4). Given that a study reported the findings from multiple time points (65), the results from other time points were included as sensitivity analysis and showed stable results 8 weeks [− 1.35(− 1.80, − 0.89)] and 12 weeks [−1.30(− 1.85, − 0.75)]. The pooled SMD value of psychological status remained stable regardless of the exclusion of any single study (Supplementary Figure S3).

## 4 Discussion

This systematic review and meta-analysis provides the first comprehensive overview of the key features and effectiveness of interventions for SSIRBs among Chinese adolescents. The earliest included studies began in 2012 and were geographically distant from inland areas, with the duration of the intervention ranging from 3 to 20 weeks. Thirteen articles were published between 2019 and 2022, which corresponded to the onset of the COVID-19 pandemic (79). Therefore, the pandemic lockdown policy captured mental health workers’ attention toward SSIRBs among adolescents.

### 4.1 Interventions for SSIRBs

The psychosocial interventions applied in Chinese studies accounted for 5 out of the 10 major categories of psychological interventions (51). Similar to the outcomes of numerous interventions targeting SSIRBs (80–84), our research also showed that psychosocial therapy was effective. A previous study showed that counseling could reduce the occurrence of SAs (85), which was similar to the findings of our study that counseling could reduce self-injury scores. In addition, our study showed that 13 out of the 19 included studies involved CBT, which was considered as the most effective intervention toward suicide reattempts (86). A systematic review among adolescents also suggested that CBT was the only intervention that could effectively reduce the risk of SIB (83). CBT focuses on an individual’s psychological and behavioral patterns and helps them understand the negative factors of SSIRBs, reduce their paranoid thoughts, reshape their perception of personal control, and ultimately achieve the goal of reducing the recurrence of SSIRBs (51, 55). Our study also found that combination therapies, including those with CBT, were more commonly used than single therapy, especially combinations of CBT and psychotherapy (31, 32, 61, 62, 64, 66, 69, 70, 72). Consistent with our study, an early study revealed that CBT combined with psychotherapy could reduce the recurrence of self-injurious behavior over a long follow-up period (87, 88). Therefore, we believe that CBT is the most widely used intervention for treating Chinese adolescents with SSIRBs.

To date, few studies in other countries have applied activity-based therapy or systemic interventions for SSIRBs, which have been applied in Chinese adolescents (59, 65, 67, 71, 74). Other intervention approaches include relationship-based interventions, psychoeducation, group work with children, peer mentoring, and intensive service models. However, no research has proven the effectiveness of these five interventions on SSIRBs among Chinese adolescents. Therefore, more attention should be given to the evaluation of the application of these interventions in the Chinese population in future.

### 4.2 Meta-analysis for NSSI

Our study revealed that psychosocial interventions for NSSI were effective, especially in the short term. Significant short-term effects of psychosocial interventions were often reported (89–91) because they immediately restored patients’ psychophysiological balance. Although long-term psychosocial interventions were also effective in our study, the effects were attenuated compared with those of short-term interventions, which might have been affected by the number of included studies and the nature of the psychosocial intervention. Meta-regression analyses found that psychosocial interventions might be more effective for small samples and male populations. On the one hand, the intervention form of psychosocial interventions (e.g., CBT) makes it easier to implement and evaluate in a small sample. On the other hand, individual interventions can provide subjects with more attention and improve effectiveness (92, 93). Due to the influence of social and cultural factors and sex roles, men are more likely to suppress and conceal their mental health problems, and men are inclined to participate more actively in the CBT treatment process by providing clear goals, solving problems, and establishing specific strategies (94). Subgroup analysis revealed that the efficacy was significantly greater immediately after the intervention compared with 1 month after the intervention. The pattern of significant short-term effectiveness was generally reflected in psychosocial interventions (95–98). Future studies should focus on assessing the long-term effects of interventions to understand their lasting impact on individuals. This can help evaluate the effectiveness and sustainability of interventions, providing evidence-based decision-making for tailored interventions.

### 4.3 Implications for future interventions

The findings from our study demonstrated that both offline and face-to-face practices were adopted in traditional interventions, indicating that CBT could significantly reduce depression and anxiety scores. In addition, CBT combined with psychotherapy could significantly reduce feelings of despair in adolescents (31, 60–62, 72). Currently, numerous innovative methods and technologies are continuously emerging, such as virtual reality, mobile applications, and online intervention platforms (99) and have been applied to improve mental health (21, 99, 100). Early studies identified that SI in adults was improved after digital interventions (21, 100). However, one study using an online intervention found that the impact of e-CBT on anxiety and despair in individuals with SI was not significant (99). Therefore, the efficacy of digital interventions for SSIRBs is still unknown. The powerful features of ChatGPT provide researchers with new opportunities (101, 102), but ethical issues also need to be considered (103). Future studies can benefit from multidisciplinary research methods that integrate knowledge from disciplines such as psychology and biology to comprehensively understand the causes of SSIRBs and intervention potential (104). Multidisciplinary approaches offer interdisciplinary perspectives and encourage the development of a deep understanding and effective interventions for SSIRBs (105).

### 4.4 Strengths and limitations

A comprehensive literature search and complex statistical analyses were carried out. Furthermore, this review not only outlined the characteristics of psychosocial interventions for SSIRBs but also performed a meta-analysis of interventions for NSSI and filled the research gap on psychosocial interventions for SSIRBs among Chinese adolescents. Our research also disseminates the results provided by researchers in China to the international community. As an alternative to drug therapy, psychosocial therapy could not only avoid the hidden dangers of insufficient evidence regarding efficacy and safety but also reduce the occurrence of many adverse reactions (106). This helps to optimize resource allocation and improve intervention effectiveness. Nevertheless, some limitations should be acknowledged. First, owing to the limited number of long-term studies, the effectiveness of the research needs to be cautiously interpreted. Second, the quality of research needs to be improved. Last, as all the reviewed studies were from mainland China, these findings may not be generalizable to other ethnicities.

## 5 Conclusion

In conclusion, our review systematically summarized the key characteristics and effectiveness of existing psychosocial interventions for SSIRBs among Chinese adolescents. CBT is the most widely used intervention for treating Chinese adolescents with NSSI. Short-term psychosocial interventions for NSSI were significantly effective in reducing self-injurious behavior scores, especially in the immediate postintervention period. More favorable treatment responses could be observed in both male and small samples. Future interventions and research should prioritize individualization, innovation, long-term outcome tracking, multidisciplinary approaches, and international collaborations.

## Author contributions

JL: Formal analysis, Writing – original draft, Writing – review & editing. WG: Methodology, Software, Writing – review & editing. NY: Data curation, Writing – review & editing. WP: Data curation, Writing – review & editing. GL: Methodology, Project administration, Writing – review & editing. WR: Methodology, Project administration, Resources, Supervision, Writing – review & editing. ZW: Data curation, Writing – review & editing.
