# Supplementary material for: Psychosocial interventions for suicidal and self-injurious-related behaviors among adolescents: a systematic review and meta-analysis of Chinese practices
Source: Front Public Health. 2023 Dec 18;11:1281696. doi: 10.3389/fpubh.2023.1281696 (PMC10757980; doi:10.3389/fpubh.2023.1281696)
Supplement: Supplementary file 1 [file Data_Sheet_1.docx]

| **Supplemental Table 1. Assessment of study quality characteristics** |
| --- |
| \| Clinical Controlled Trials ^#^ \| \| \| \| \| \| \| \| \| \| \| \| \| \| \| \| \| --- \| --- \| --- \| --- \| --- \| --- \| --- \| --- \| --- \| --- \| --- \| --- \| --- \| --- \| --- \| --- \| \| Author, year \| 1.  Described as an RCT \| 2.  Randomization method \| 3.  Concealed treatment allocation \| 4.  Blinded participants \| 5.  Blinded assessors \| 6.  Similar groups  at baseline \| 7.  Low drop-out rate \| 8.  Low differential drop-out rate between groups \| 9.  High adherence to the intervention protocols \| 10.  Avoided other or similar interventions \| 11.  Valid and reliable measures \| 12.  Sufficient sample size \| 13.  Prespecified outcomes or subgroup analyses \| 14.  Consistent randomized group \| Total score \| \| Xue YW,  2022 \| 0 \| 0 \| 0 \| 0 \| 0 \| 1 \| 1 \| 1 \| 1 \| 1 \| 1 \| NR \| 0 \| 1 \| 7 \| \| Xue Y,  2022 \| 0 \| 0 \| 0 \| 0 \| 0 \| 1 \| 1 \| 1 \| 1 \| 1 \| 1 \| NR \| 0 \| 1 \| 7 \| \| Rong J,  2020 \| 0 \| 0 \| 0 \| 0 \| 0 \| 1 \| 1 \| 1 \| 1 \| 1 \| 1 \| NR \| 0 \| 1 \| 7 \| \| Li L,  2012 \| 0 \| 0 \| 0 \| 0 \| 0 \| 1 \| 0 \| 1 \| 1 \| 1 \| 1 \| NR \| 1 \| 1 \| 7 \|  1. Was the study described as a randomized trial, a randomized clinical trial, or an RCT? 2. Was the method of randomization adequate (i.e., use of randomly generated assignment)? 3. Was the treatment allocation concealed (so that assignments could not be predicted)? 4. Were study participants and providers blinded to treatment group assignment? 5. Were the people assessing the outcomes blinded to the participants' group assignments? 6. Were the groups similar at baseline on important characteristics that could affect outcomes? 7. Was the overall drop-out rate from the study at endpoint 20% or lower of the number allocated to treatment? 8. Was the differential drop-out rate (between treatment groups) at endpoint 15 percentage points or lower? 9. Was there high adherence to the intervention protocols for each treatment group? 10. Were other interventions avoided or similar in the groups? 11. Were outcomes assessed using valid and reliable measures, implemented consistently across all study participants? 12. Did the authors report that the sample size was sufficiently large to be able to detect a difference in the main outcome between groups with at least 80% power? 13. Were outcomes reported or subgroups analyzed prespecified (i.e., identified before analyses were conducted)? 14. Were all randomized participants analyzed in the group to which they were originally assigned? |
| \| Pre- & post-test studies ^#^ \| \| \| \| \| \| \| \| \| \| \| \| \| \| \| \| --- \| --- \| --- \| --- \| --- \| --- \| --- \| --- \| --- \| --- \| --- \| --- \| --- \| --- \| --- \| \| Author, year \| 1.  Clear study question \| 2. Prespecified selection criteria \| 3. Representative sample \| \| 4.  Eligible samples all enrolled \| 5.  Sufficient sample size \| 6.  Clear description & consistent delivery \| 7.  Valid & reliable outcome measures \| 8.  Blinded assessor \| 9.  Low loss to follow-up \| 10.  Appropriate statistical methods \| 11.  Outcome measured multiple times \| 12.  Individual level data considered in analyses \| Total  score \| \| Xie HT, 2014 \| 1 \| 1 \| 0 \| 1 \| \| NR \| 1 \| 1 \| 0 \| 1 \| 1 \| 1 \| NR \| 8 \| \| Liu JT, 2013 \| 1 \| 1 \| 1 \| 1 \| \| NR \| 1 \| 1 \| 0 \| 1 \| 1 \| 0 \| NR \| 8 \| \| Lin YT, 2019 \| 1 \| 1 \| 0 \| 1 \| \| NR \| 1 \| 1 \| 0 \| 1 \| 0 \| 0 \| NR \| 6 \| \| Chang XD, 2015 \| 1 \| 1 \| 1 \| 1 \| \| NR \| 1 \| 1 \| 0 \| 1 \| 1 \| 0 \| NR \| 8 \| \| Li JC, 2016 \| 1 \| 1 \| 0 \| 1 \| \| NR \| 1 \| 1 \| 0 \| 1 \| 1 \| 1 \| NR \| 8 \|  1. Was the study question or objective clearly stated? 2. Were eligibility/selection criteria for the study population prespecified and clearly described? 3. Were the participants in the study representative of those who would be eligible for the test/service/intervention in the general or clinical population of interest? 4. Were all eligible participants that met the prespecified entry criteria enrolled? 5. Was the sample size sufficiently large to provide confidence in the findings? 6. Was the test/service/intervention clearly described and delivered consistently across the study population? 7. Were the outcome measures prespecified, clearly defined, valid, reliable, and assessed consistently across all study participants? 8. Were the people assessing the outcomes blinded to the participants' exposures/interventions? 9. Was the loss to follow-up after baseline 20% or less? Were those lost to follow-up accounted for in the analysis? 10. Did the statistical methods examine changes in outcome measures from before to after the intervention? Were statistical tests done that provided p values for the pre-to-post changes? 11. Were outcome measures of interest taken multiple times before the intervention and multiple times after the intervention (i.e., did they use an interrupted time-series design)? 12. If the intervention was conducted at a group level (e.g., a whole hospital, a community, etc.) did the statistical analysis take into account the use of individual-level data to determine effects at the group level? |
| \| Randomized Controlled Trials ^¶^ \| \| \| \| \| \| \| \| \| \| --- \| --- \| --- \| --- \| --- \| --- \| --- \| --- \| --- \| \| Author, year \| 1.  Described as an RCT \| 2.  Appropriate randomization sequence method \| 3.  Described as double blind study \| Appropriate double blinding method \| Description of withdrawals and dropouts \| Inappropriate description of the method to generate the sequence of randomisation \| Inappropriate description of double blind \| Total  Score \| \| Yang RL, 2021 \| 1 \| 1 \| 0 \| 0 \| 0 \| 0 \| / \| 2 \| \| Xie HX, 2022 \| 1 \| 0 \| 0 \| 0 \| 0 \| / \| / \| 1 \| \| Wang YP, 2022 \| 1 \| 1 \| 0 \| 0 \| 1 \| 0 \| / \| 3 \| \| Su XY, 2022 \| 1 \| 1 \| 0 \| 0 \| 0 \| 0 \| / \| 2 \| \| Huang J, 2022 \| 1 \| 0 \| 0 \| 0 \| 0 \| / \| / \| 1 \| \| Li BC, 2016 \| 1 \| 0 \| 0 \| 0 \| 0 \| / \| / \| 1 \| \| Du WL, 2022 \| 1 \| 0 \| 0 \| 0 \| 0 \| / \| / \| 1 \| \| Ding D, 2021 \| 1 \| 1 \| 0 \| 0 \| 0 \| 0 \| / \| 2 \| \| Chen G, 2022 \| 1 \| 1 \| 0 \| 0 \| 0 \| 0 \| / \| 2 \| \| Xia S, 2022 \| 1 \| 1 \| 0 \| 0 \| 0 \| 0 \| / \| 2 \| |

1.Was the study described as randomized (this includes words such as randomly, random, and randomization)? (yes/no); 2. Was the method used to generate the sequence of randomization described and appropriate (table of random numbers, computer-generated, etc.)? (yes/no); 3. Was the study described as double blind? (yes/no); 4. Was the method of double blinding described and appropriate (identical placebo, active placebo, dummy, etc.)? (yes/no); 5. Was there a description of withdrawals and dropouts? (yes/no); 6. Deduct one point if the method used to generate the sequence of randomization was described and it was inappropriate (e.g. patients were allocated alternately, or according to date of birth, hospital number, etc.).(Described but inappropriate = -1, Described and appropriate = 0); 7. Deduct one point if the study was described as double blind but the method of blinding was inappropriate (e.g. comparison of tablet vs. injection with no double dummy).(Described but inappropriate = -1, Described and appropriate = 0)

*Notes*

^#^ National Heart Lung and Blood Institute. (2021). Study Quality Assessment Tools. Retrieved August 4, 2022, from https://www.nhlbi.nih.gov/health-topics/study-quality-assessment-tools.

^¶^ Jadad AR, Moore RA, Carroll D, et al. Assessing the quality of reports of randomised clinical trials: is blinding necessary? Controlled Clinical Trials 1996; 17(1): 1-12

**Supplemental Table 2. Meta-regression and subgroup analyses of effectiveness at short-term post-intervention**

| **Category** | **Variable** | **slope** | **SE** | **Z** | ***P*** | **95%CI** | |
| --- | --- | --- | --- | --- | --- | --- | --- |
| Meta-regression analysis | Sample size | 0.068 | 0.023 | 2.914 | **0.004** | 0.022 | 0.113 |
|  | Mean age | 1.194 | 1.151 | 1.037 | 0.300 | -1.063 | 3.451 |
|  | Sex ratio (F/M) | 1.096 | 0.187 | 5.848 | **<0.001** | 0.728 | 1.463 |
| **Subgroup analysis** | **Category** | **Sample Size** | **Effect**  **Size** | **95% CI** | | **I^2^** | ***P* across subgroup** |
| Assessment  period | Immediate post-intervention (4) | 190 | -2.800 | -4.050 | -1.550 | 95.031 | **<0.001** |
|  | Less than  1 month (2) | 366 | -0.494 | -0.783 | -0.205 | 0 |  |
| Scales | ANSAQ (2) | 216 | -0.860 | -1.140 | -0.581 | 43.869 | 0.360 |
|  | QMSSB/ASHS (2) | 220 | -1.170 | -2.755 | 0.416 | 96.548 |  |
|  | Others (2) | 120 | -5.003 | -10.881 | 0.875 | 97.246 |  |
| Regions | Eastern of China (3) | 306 | -1.136 | -2.013 | -0.260 | 91.982 | 0.068 |
|  | Western of China (3) | 250 | -3.200 | -5.232 | -1.168 | 97.039 |  |
| Definition of NSSI | DSM-V (2) | 140 | -4.161 | -11.715 | 3.393 | 98.393 | 0.757 |
|  | Hospitalization (2) | 210 | -1.311 | -2.622 | 0 | 94.612 |  |
|  | Others (2) | 206 | -1.530 | -2.554 | -0.507 | 89.682 |  |

Notes:

ANSAQ, Adolescent Non-suicidal Self-injury Assessment Questionnaire; ASHS, Adolescent Self Harm Scale; CI, Confidence interval; DSM-V, Diagnostic and statistical manual of mental disorders (Fifth edition); NSSI, Non-suicidal self-injury; QMSSB, Questionnaire for Middle School Students’ Behavior; SE, Standard Error.

1. **Wanfang**


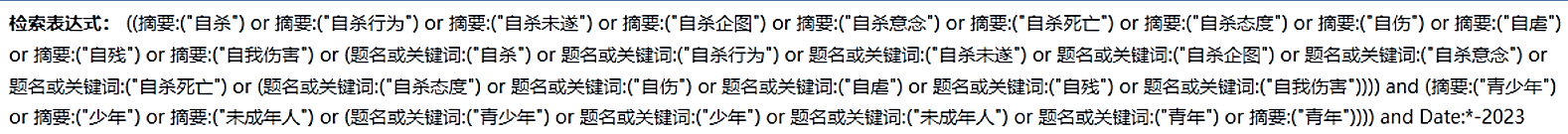


1. **CNKI**

**
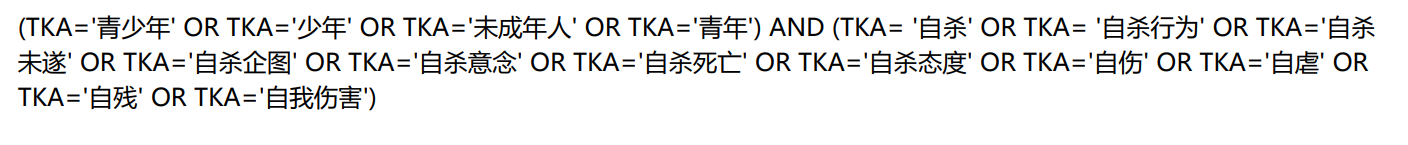
**

1. **SinoMed**

**
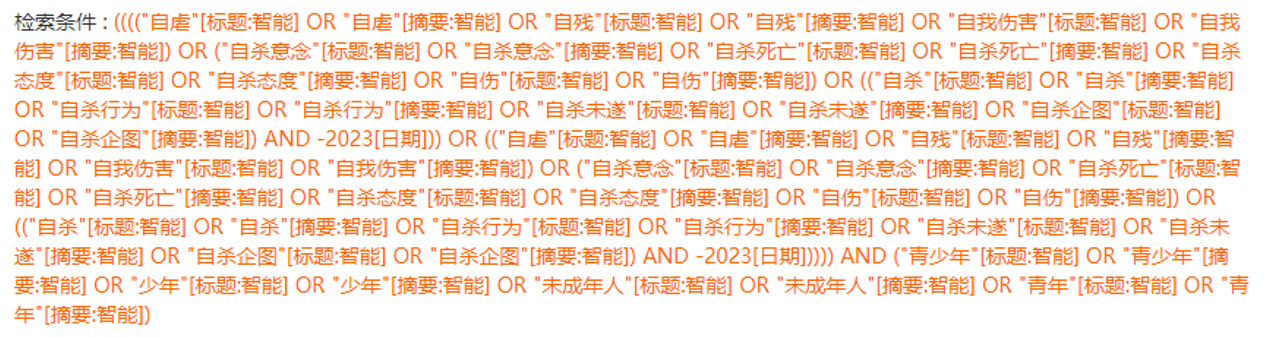
**

1.
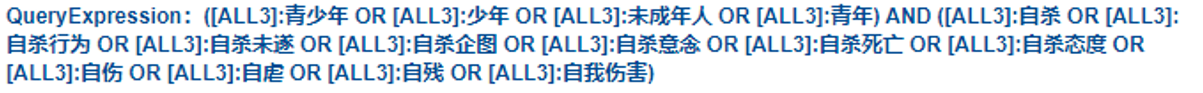
**CEPS**
2. **CINAHL and PsycINFO**

**
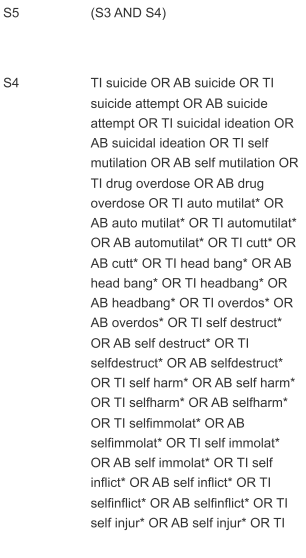
**

**
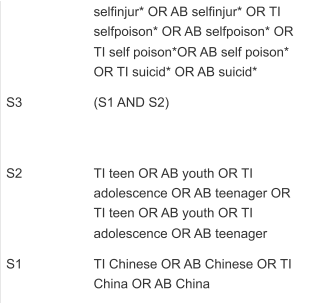
**

**F-** **Cochrane Library**

**
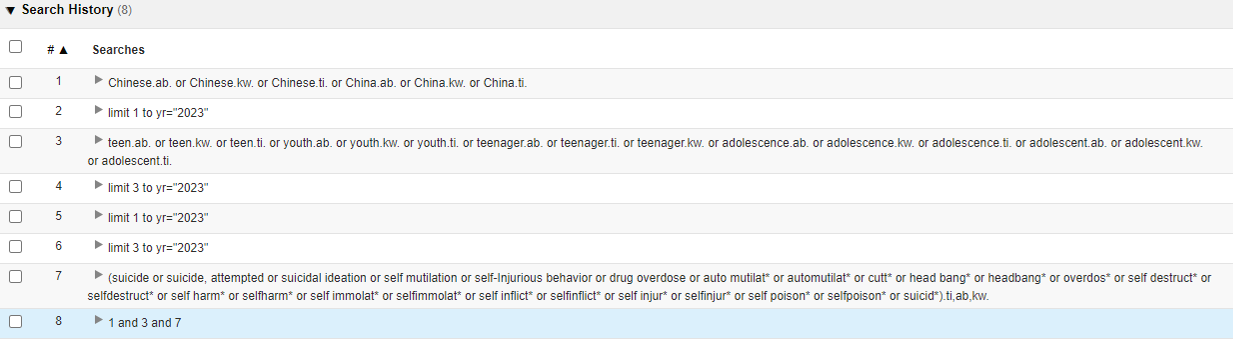
**

**G-EMBASE**

**
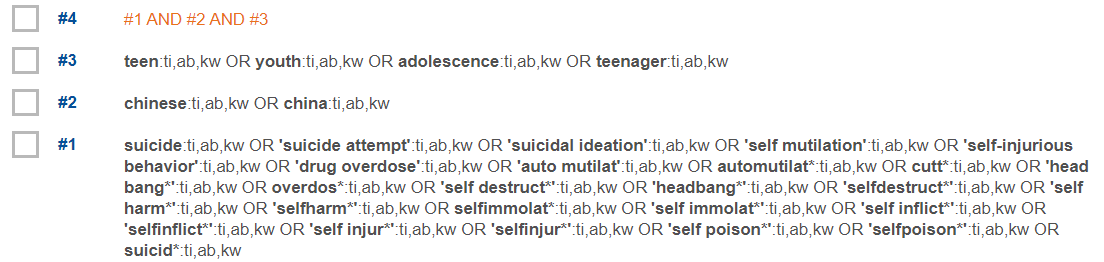
**

**H-Web of Science**

**
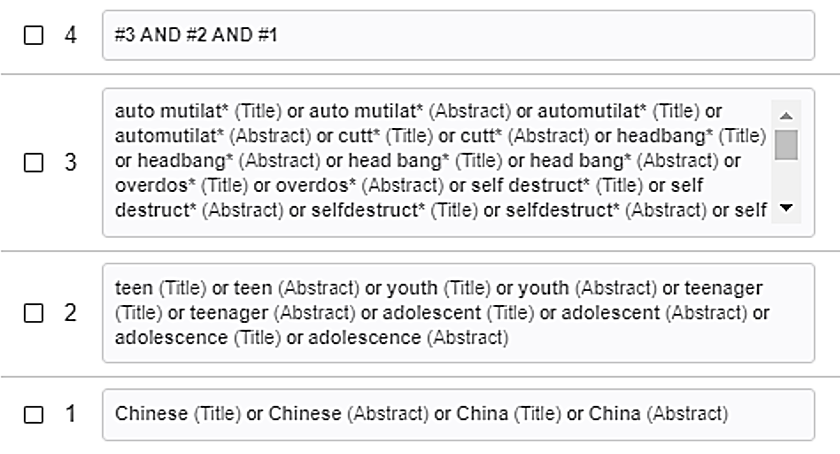
**

**
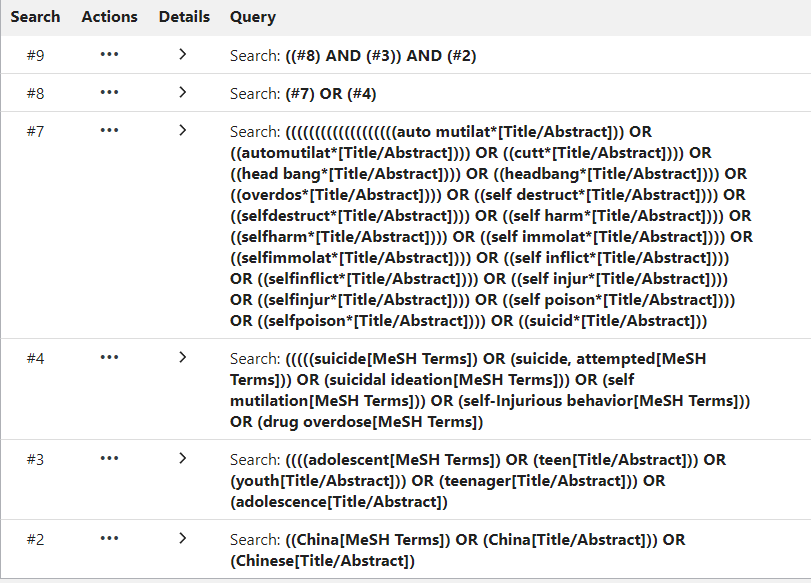
I-PubMed**

**J-Clinical Trial and ScienceDirect based on keywords.**

**Supplemental Figure 1. Search strategy**

**
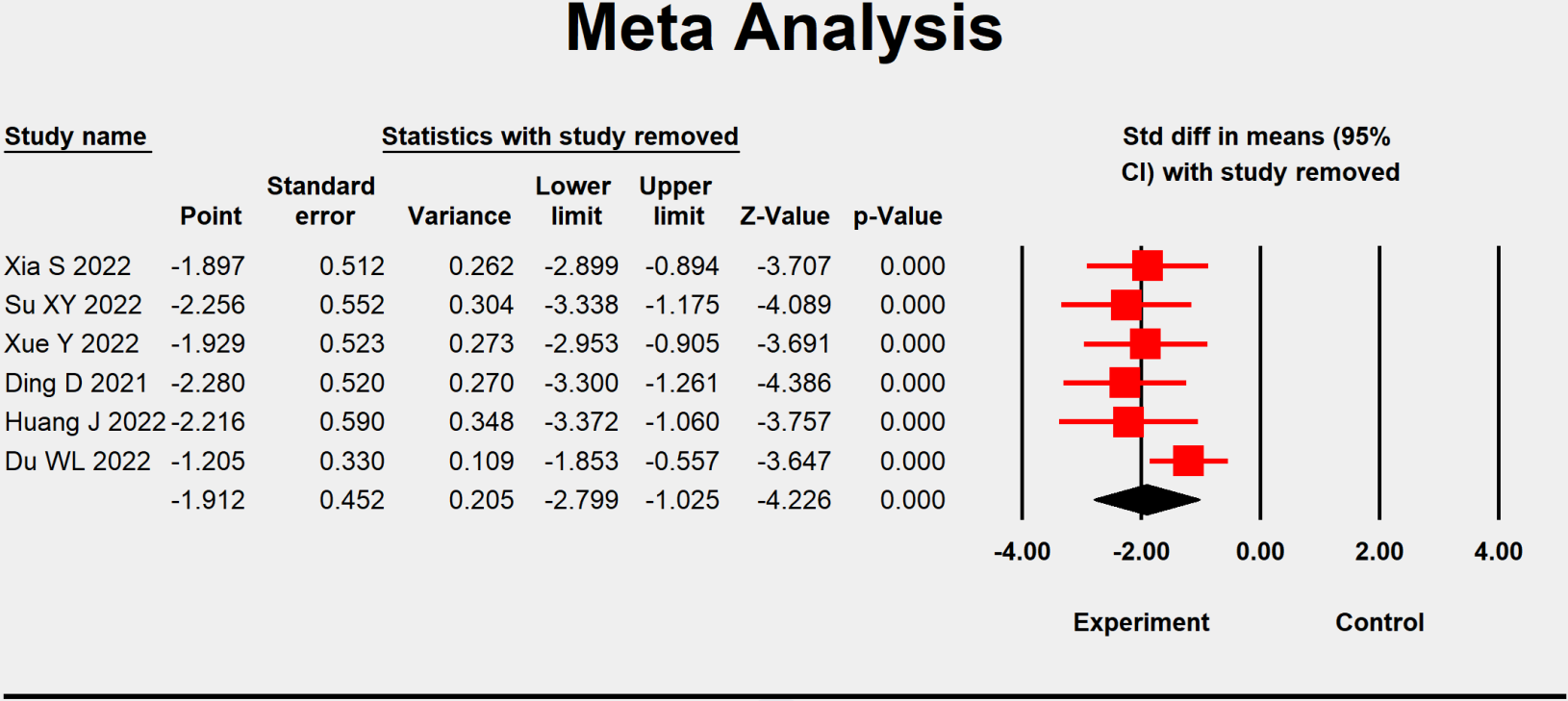
**

**Supplemental Figure 2. Sensitivity analysis - short term**


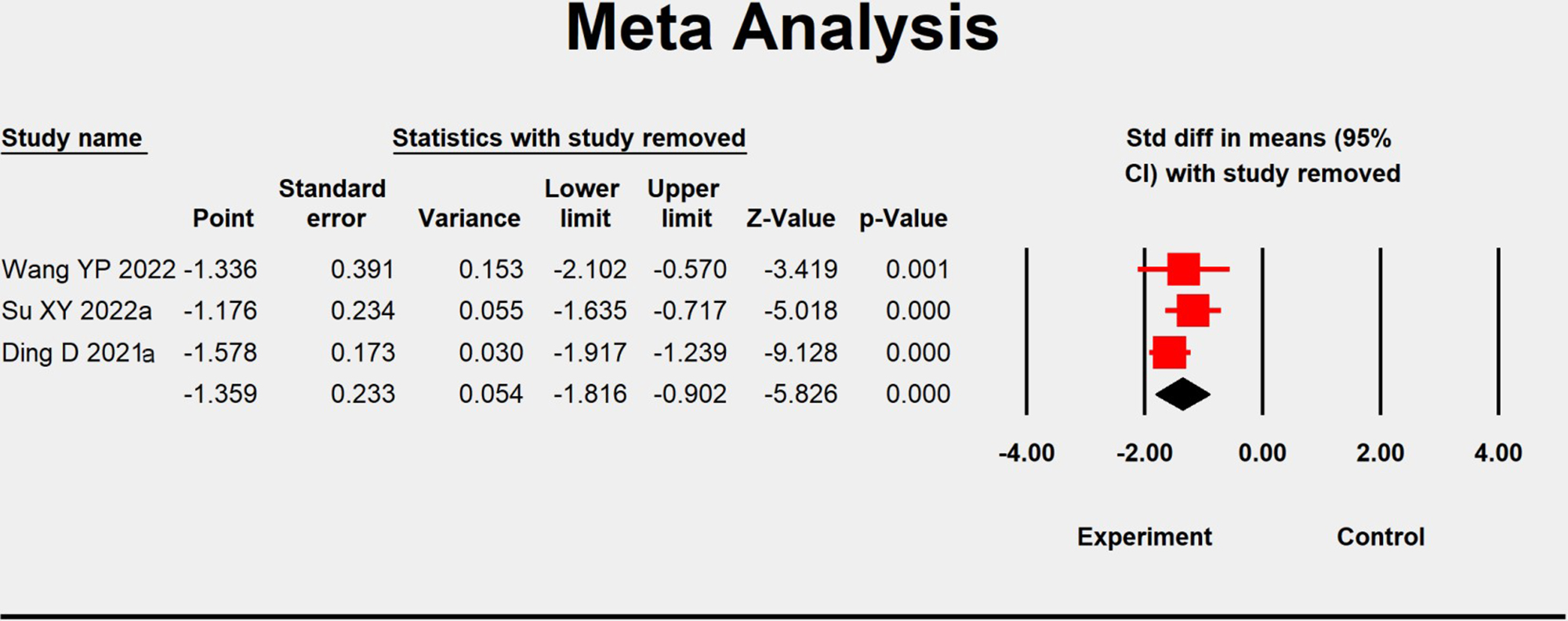


**Supplemental Figure 3. Sensitivity analysis - long term**

Note:

Su XY 2022a means the evaluation period: 6 weeks after intervention.

Ding D 2021a means the evaluation period: 16 weeks after intervention.
